# Novel, Green, Fast, and Scalable Method for Producing Dendritic Mesoporous Silica Nanoparticles (DMSNs) with High Channel Accessibility

**DOI:** 10.1021/acsomega.6c02716

**Published:** 2026-07-03

**Authors:** Roberta Zanini, Sabrina Molinaro, Luca Leoncino, Simone Lauciello, Filippo Drago, Rosaria Brescia, Sergio Marras, Elti Cattaruzza, Mauro Moglianetti, Arianna Traviglia

**Affiliations:** † Istituto Italiano di Tecnologia, Center for Cultural Heritage Technology, via Adriano Olivetti, 1, Roncade, TV 31056, Italy; ‡ Department of Molecular Sciences and Nanosystems, Ca’ Foscari University of Venice, via Torino, 155, Venice 30172, Italy; § Istituto Italiano di Tecnologia, Electron Microscopy Facility, Via Morego 30, Genoa 16163, Italy; ∥ Istituto Italiano di Tecnologia Chemistry Facility, Via Morego 30, Genoa 16163, Italy; ⊥ Istituto Italiano di Tecnologia Material Characterisation Facility, Via Morego 30, Genoa 16163, Italy

## Abstract

This work provides the first experimental evidence of a fully novel and innovative green-synthesis protocol for dendritic mesoporous silica nanoparticles (DMSNs). DMSNs are widely used in various scientific fields, such as drug delivery, catalysis, and environmental remediation; however, their production still presents critical challenges, particularly with respect to sustainability for both operators and the environment. The novel method proposed here is rapid and straightforward, carried out entirely in aqueous solution using a microwave reactor operated in sealed vessels. Owing to the simplicity and strict parameter control of this approach, the roles of temperature and pressure in directing surfactant self-assembly are reported for the first time, resulting in a center-symmetric arrangement of the mesoporous structure. This method eliminates the need for organic solvents, pore modifiers, or cosurfactants, which are typically required to obtain the radial mesostructure. Moreover, the use of microwave-based reactors enables scalability through a modular setup and allows for a significant reduction in reaction time (∼10 min) and energy consumption. A comparative analysis with mesoporous silica nanoparticles (MSNs) featuring longitudinal channel structures reveals that the radial configuration of DMSNs exhibits more efficient mass transport, higher loading capacity for active species, and improved diffusion dynamics, which translate into superior performance across several applications.

## Graphical abstract

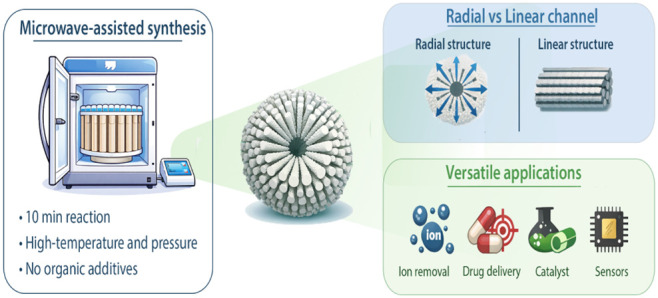

## 1 Introduction

Dendritic mesoporous silica nanoparticles (DMSNs) exhibit a unique radial-channel morphology that facilitates channel accessibility and versatile functionalization, making them highly suitable for a variety of applications.^1–4^ As their use continues to expand across these application areas, there is a growing need for more sustainable synthetic approaches that minimize environmental impact by reducing overall energy consumption and limiting the use of toxic organic solvents typically employed in their production.

Although existing synthesis methods for DMSNs provide valuable control over pore morphology, surface characteristics, and porosity, and enable tunable pore structures and radial architectures.^3,5^ However, in the specific case of DMSNs, organic solvents or pore-modifying agents are not merely optional but are typically essential to induce the formation of dendritic pore structures, as widely reported in the literature, and in the text below. This dependence limits the environmental sustainability of existing methods and contributes to their complexity and limited scalability.

Three main synthesis approaches are commonly used to produce DMSNs.^6^ The first, the emulsion synthesis method,^7–9^ relies on nanoscale droplets in microemulsions as confined spaces for particle growth, with organic swelling agents, such as toluene and cyclohexane, used to tune the pore structure and promote dendritic morphology. In 2014, Moon and Lee^10^ demonstrated that the structure of DMSNs can be controlled by modulating the water–surfactant–oil mixing ratio and introducing various cosolvents; however, this method requires careful optimization and relies on organic solvents, limiting its environmental sustainability. The second approach, biphase stratification,^11–13^ features an oil–water interface where the silica precursor diffuses gradually, enabling controlled formation of dendritic fibrous nanostructures. Yet the use of oil phases and organic solvents complicates the procedure and undermines its alignment with green chemistry principles. The third approach, the homogeneous synthesis method,^14–16^ occurs entirely in a single aqueous phase and typically employs a surfactant-assisted approach using cetyltrimethylammonium (CTA+) as the templating surfactant and the dendritic mesochannel morphology is controlled by adding a template counterion, such as tosylate (Tos−) and bromide (Br−),^14^ or small organic amines like triethanolamine.^15^ Despite these advances, existing methods generally require organic solvents or pore-modifying agents to achieve radial architectures.

To overcome these challenges, the present study introduces a surfactant-based synthesis protocol that enables the formation of dendritic pore structures without the use of additional organic solvents, pore modifiers, or organic catalysts. This represents a distinctive approach, subject to a patent, compared to existing methodologies, where such organic components are typically required to achieve dendritic morphologies. Furthermore, this new method significantly reduces synthesis time, lowering overall energy consumption and supporting its classification as a greener alternative. Since tunability is outside the scope of this study, emphasis will be placed on a simplified and more sustainable synthetic route for DMSNs. The use of a microwave reactor enables simultaneous increases in temperature and pressure within closed vessels, thereby making the synthesis rapid, scalable, and capable of producing gram-scale nanoparticles within a short reaction time. While large-scale production was not experimentally demonstrated here, microwave-assisted systems are already implemented in industrial reactors, suggesting that the approach is, in principle, scalable.

Moreover, a comprehensive comparison of the channel structures of DMSNs and conventional MSNs demonstrates the superior performance of the radial channel morphology over the longitudinal one. The enhanced amine functionalization and subsequent ion-removal capability of DMNSs underscore their superior physicochemical properties, confirming their broad applicability across diverse fields, ranging from water remediation to advanced delivery systems.

## 2 Experimental Section

### 2.1 Materials

All reagents and solvents were purchased from Sigma-Aldrich and used as received. Co­(NO_3_)_2_·6H_2_O was purchased by abcr GmbH.

### 2.2 Synthesis of MSNs and DMSNs and Their Functionalization

MSNs were synthesized following the protocol reported by Catalano and Pompa.^17^ The DMSNs with a radial channel structure were produced using a microwave-assisted synthesis with the Milestone Ethos UP and a multivessel setup (15 vessels). The protocol requires two steps. In the first step, 0.04 g of cetyltrimethylammonium bromide (CTAB) was added to 40 mL of Milli-Q water in a microwave Teflon tube. Then, 0.30 mL of 2 M NaOH, as a catalyst, was added to the water solution and stirred at 80 °C for 10 min. In the second step, 0.42 mL of tetraethyl orthosilicate (TEOS) was added dropwise to the solution without stirring. Then, the solution was placed in the microwave, and the temperature was raised to 150 °C in 5 min and kept at this temperature for 10 min under moderate stirring. By decreasing the quantity of NaOH to 0.15 mL, we obtained smaller DMSNs with reduced polydispersity.

The resulting opaque solutions were centrifuged and extensively washed by several resuspension and centrifugation cycles with a mixture of water and ethanol (EtOH). CTAB within the pores was removed via extraction with an acid solution, refluxing the NPs suspended in 160 mL of EtOH and 9 mL of hydrochloric acid (HCl, 37%) at 60 °C for 24 h. Purified NPs were then washed and collected by several centrifugations and dispersed in EtOH. The same purification protocol was applied for MSNs.

The functionalization with amine groups was performed according to the protocol described by Wang et al.^18^ for both MSNs and DMSNs, but using ethanol instead of toluene due to toxicity concerns: 500 mg of nanoparticles were dispersed in 50 mL of EtOH, and 0.8 mL of APTES was added. The solution was stirred for 20 h at 80 °C, and the resulting solution was washed via several centrifugations.

### 2.3 Morphological and Physicochemical Characterization of MSNs and DMSNs

Transmission electron microscopy (TEM) and scanning electron microscopy (SEM) were employed to investigate the particle dimensions, morphology, shape, and channel structures. Bright-field TEM imaging was performed with a Tecnai G^2^ F20 TWIN TMP microscope on samples drop-cast onto continuous amorphous carbon film on Cu grids. The SEM images reported here were acquired on the same samples using the in-lens secondary electron detector of a Zeiss Gemini SEM 560, operated at 7 kV. High-angle-annular dark field-scanning TEM (HAADF-STEM) imaging and energy dispersive X-ray spectroscopy (EDXS) analyses were carried out on an image-corrected JEOL JEM-2200FS TEM, operated at 200 kV, equipped with a Bruker XFlash-5060 silicon-drift detector. For the latter analyses, the samples were drop-cast onto holey carbon-coated Cu grids. The EDXS elemental quantification presented here was obtained on spectra collected on particles suspended on holes, to avoid possible Si contribution from the support film. Quantification was performed using the Cliff-Lorimer method on the Kα peaks of Si and Co. The channel geometry, periodicity, and inner-diameter distribution were extracted from BF-TEM and HAADF-STEM images and their corresponding fast Fourier transforms (FFTs). The FFTs have been azimuthally integrated and background-subtracted using PASAD.^19^

X-ray diffraction (XRD) was employed to evaluate the structural order and confirm the amorphous nature of the synthesized DMSNs. The analysis was recorded on a Malvern-PANalytical 3rd generation Empyrean X-ray diffractometer, equipped with a 1.8 kW Cu X-ray tube (λCuKα = 0.15406 nm) operating at 40 kV and 45 mA, W/Si elliptic focusing mirror, PIXcel*3D* area detector. The samples were placed between two layers of 5 μm-thick Mylar foil. The diffraction patterns were collected at room temperature in transmission geometry using a spinner sample stage (rotation speed = 2 rps).

The physical and chemical properties of the nanoparticles were thoroughly characterized using a combination of analytical techniques. N_2_ adsorption–desorption analysis was employed using a Micromeritics ASAP 2010 Micromeritics TriStar II Plus system (Micromeritics Instrument Inc., USA) before and after functionalization. The Brunauer–Emmett–Teller (BET) and Brunauer–Joyner–Halenda (BJH) methods were applied to evaluate surface area and pore volume and size, respectively. Approximately 100 ± 10 mg of samples were degassed overnight at 80 °C under vacuum as a pretreatment for physisorption measurements. Mass loss upon degassing was evaluated by weighing the samples before and after treatment.

ζ-Potential analysis was conducted to assess the surface charge and verify the success of surface functionalization. Measurements were carried out using a Zetasizer Lab system (Malvern Panalytical), using a concentration of 0.1 mg/mL of NPs in a Milli-Q water solution at a pH range of 2 to 9 obtained by adding acetic acid and NaOH.

In addition, thermogravimetric analysis (TGA) was used to quantify the weight loss due to the degradation of the organic content, confirming the presence of amine groups introduced during functionalization. This thermal analysis was performed under a nitrogen atmosphere (50 mL/min) using a TGA Q500 instrument (TA Instruments), with samples heated from 30 to 700 °C at a rate of 10 °C/min to monitor decomposition profiles and mass loss.

### 2.4 Cobalt Removal from Aqueous Solution and Loading into the Nanoparticles

For the ion loading test, 1 mg/mL of DMSN–NH_2_ or MSN–NH_2_ was added to an aqueous solution where cobalt nitrate was dissolved at a concentration of 0.5 mg/mL, in a total volume of 2 mL. At various time intervals, the supernatant and NPs were separated by centrifugation, and the residual Co^2+^ concentration in the supernatant and the amount loaded onto the NPs were quantified by inductively coupled plasma optical emission spectrometry (ICP-OES). Elemental analysis of the samples was performed using inductively coupled plasma optical emission spectrometry (ICP-OES) with an iCAP 7600 DUO spectrometer (Thermo Fisher Scientific). For each measurement, 500 μL aliquots were taken from the liquid samples. Each aliquot was treated with 1 mL of nitric acid (HNO_3_, 67–69%, Trace Metal grade). Samples requiring silicon quantification were pretreated with 100 μL of HF before the addition of HNO_3_. Prior to analysis, each sample was diluted with Milli-Q water to a final volume of 10 mL and finally filtered using a 0.45 μm PTFE membrane filter. The ICP-OES operating conditions for elemental quantification were as follows: auxiliary gas flow rate of 0.50 L min^–1^, cooling gas flow rate of 12 L min^–1^, nebulizer gas flow rate of 0.50 L min^–1^, nebulizer gas pressure of 180 kPa, RF power of 1150 W, and a peristaltic pump speed of 50 rpm. Elemental calibration was performed using a four-point calibration curve (excluding the blank) at concentrations of 0.01, 0.1, 1, and 10 ppm. The ICP-OES analysis exhibited a systematic error of approximately 5%.

## 3 Results and Discussion

### 3.1 Microwave-Assisted, Green, and Fast Synthesis of DMSNs

The novel approach proposed here for synthesizing silica nanoparticles with a center-symmetric channel arrangement is a green, simple, and fast method. It overcomes major limitations associated with the use of organic pore modifiers and organic catalysts, achieving a dendritic morphology by combining temperatures above the boiling point (150 °C) with the resulting increase in pressure within the closed reaction containers.

SEM images of DMSNs in Figure 1a–c show the uniform spherical shape and pore density. TEM images in Figure 1d–f clearly reveal the radial mesoporous structure of the DMSNs, highlighting, in particular, the shape of the channels: unlike the mixed conical-cylindrical shape shown in previous studies,^20^ the channels in this case exhibit mostly cylindrical shape throughout the particle volume, with a characteristic inner diameter of about 2 nm. However, as particle growth proceeds, new walls nucleate within existing channels in order to keep the inner diameter constant from the particle core to the surface.

**Figure 1:**
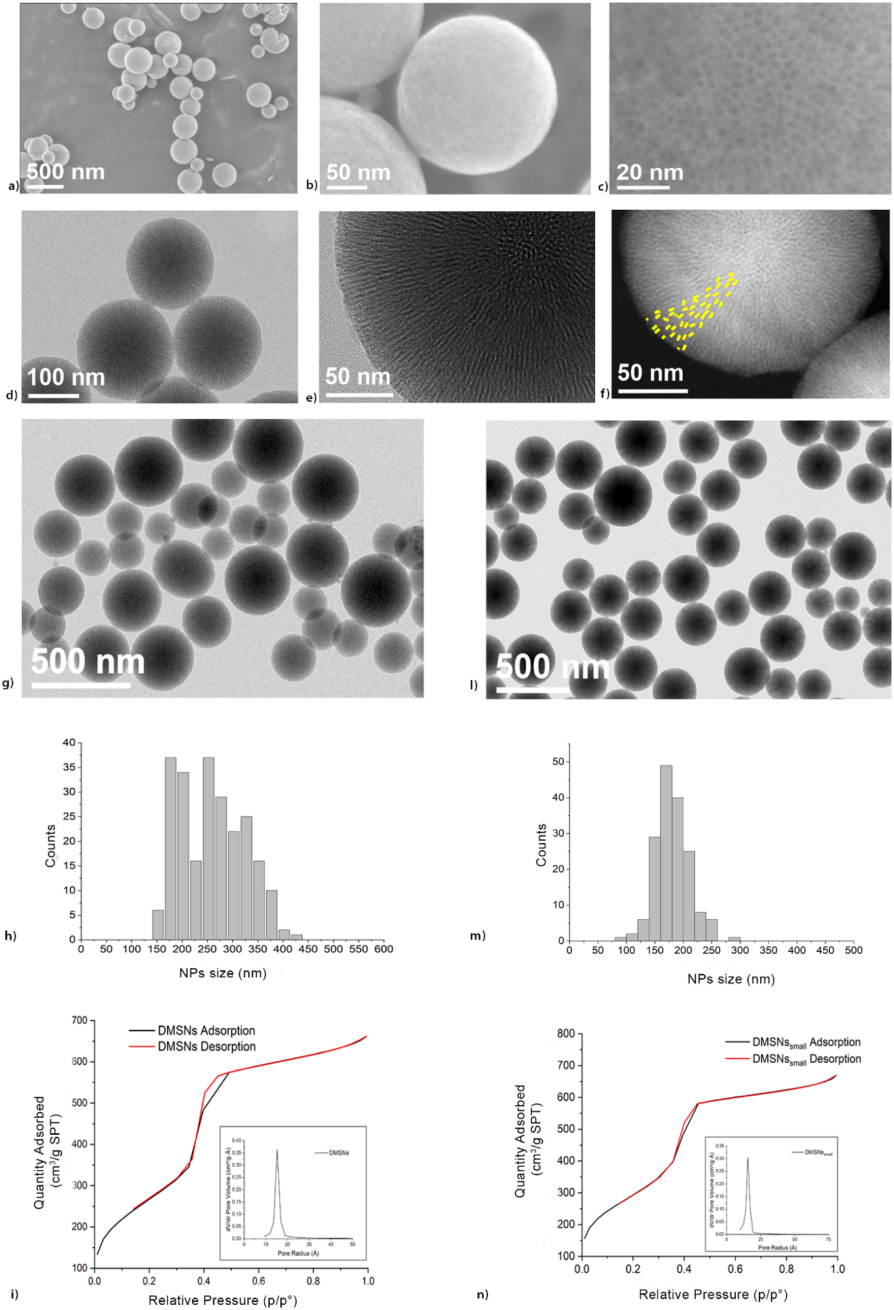
(a) and (b) SEM images of DMSNs at different magnifications, with (c) a high magnification view of the surface pore structure; (d) and (e) BF-TEM images of few DMSNs, clearly showing the radial mesoporous structure and (f) HAADF-STEM image of a portion of a DMSN, with few channel walls traced (yellow dashed lines). (g) and (l) BF-TEM images, (h) and (m) size distribution (performed considering ∼300 nanoparticles in BF-TEM images), (i) and (n) N_2_ adsorption–desorption curves of DMSNs obtained with 0.30 and 0.15 mL of NaOH, respectively.

The protocol is highly versatile, allowing tuning of DMSNs size by simply adjusting the catalyst concentration (NaOH). For instance, at low NaOH concentration, particles with a diameter centered at ∼170 nm and a low polydispersity were obtained (Figure 1l and m). By increasing the NaOH concentration, the nanoparticles’ size and, consequently, their polydispersity increase, resulting in a multimodal particle size distribution spanning from 100 to 300 nm (Figure 1g and h). This broader size distribution may be attributed to the synthesis setup. Specifically, TEOS addition is performed outside the microwave system, dropwise, without stirring or temperature control, which can lead to slight variations in nucleation and growth kinetics. These effects, combined with the faster hydrolysis and condensation rates at higher NaOH concentrations, may contribute to the observed DMSN polydispersity.

From a physical point of view, the DMSNs exhibit a high specific surface area (approximately 1000 m^2^/g, as shown in Figure 1i and n). The adsorption–desorption isotherms follow a typical type IV isotherm, which is characteristic of mesoporous structures.^24,25^

The synthesis method presented here does not rely on cosurfactants, organic solvents, or pore modifiers, yet successfully yields DMSNs with a high surface area, demonstrating that a highly accessible radial morphology can be obtained with a simplified, organic solvent-free protocol. This approach overcomes the need for toxic organic compounds in biphasic solutions or cosurfactants in homogeneous synthesis, addressing major limitations reported in the literature (Table SI1 in SI) while providing an eco-friendly alternative for both operators and the environment. The method also aligns closely with green chemistry principles, without compromising control over the nanoparticles’ physicochemical properties.

Moreover, the microwave-assisted synthesis significantly reduces reaction time while maintaining strict control over particle characteristics. Unlike conventional protocols, which often require several hours, this approach enables the production of 0.8 g of DMSNs in less than 15 min. This rapid synthesis not only enhances reproducibility and scalability (overcoming a key limitation of traditional methods)^21^ but also facilitates direct translation from laboratory research to large-scale applications.

### 3.2 The Role of Pressure in the Self-Assembly of Surfactant for the Formation of Radial Structure Channels

The formation of the dendritic channel structure is achieved by raising the temperature above the solvent’s boiling point in sealed vessels, which results in a concomitant increase in pressure in the reaction container. While reports present in the literature on CTAB phase diagrams primarily emphasize the role of surfactant concentration and temperature in governing the properties of a CTAB/water system,^22,23^ in this work, we discovered that pressure also plays a crucial role in driving the CTAB self-assembly (Figure 2).

**Figure 2:**
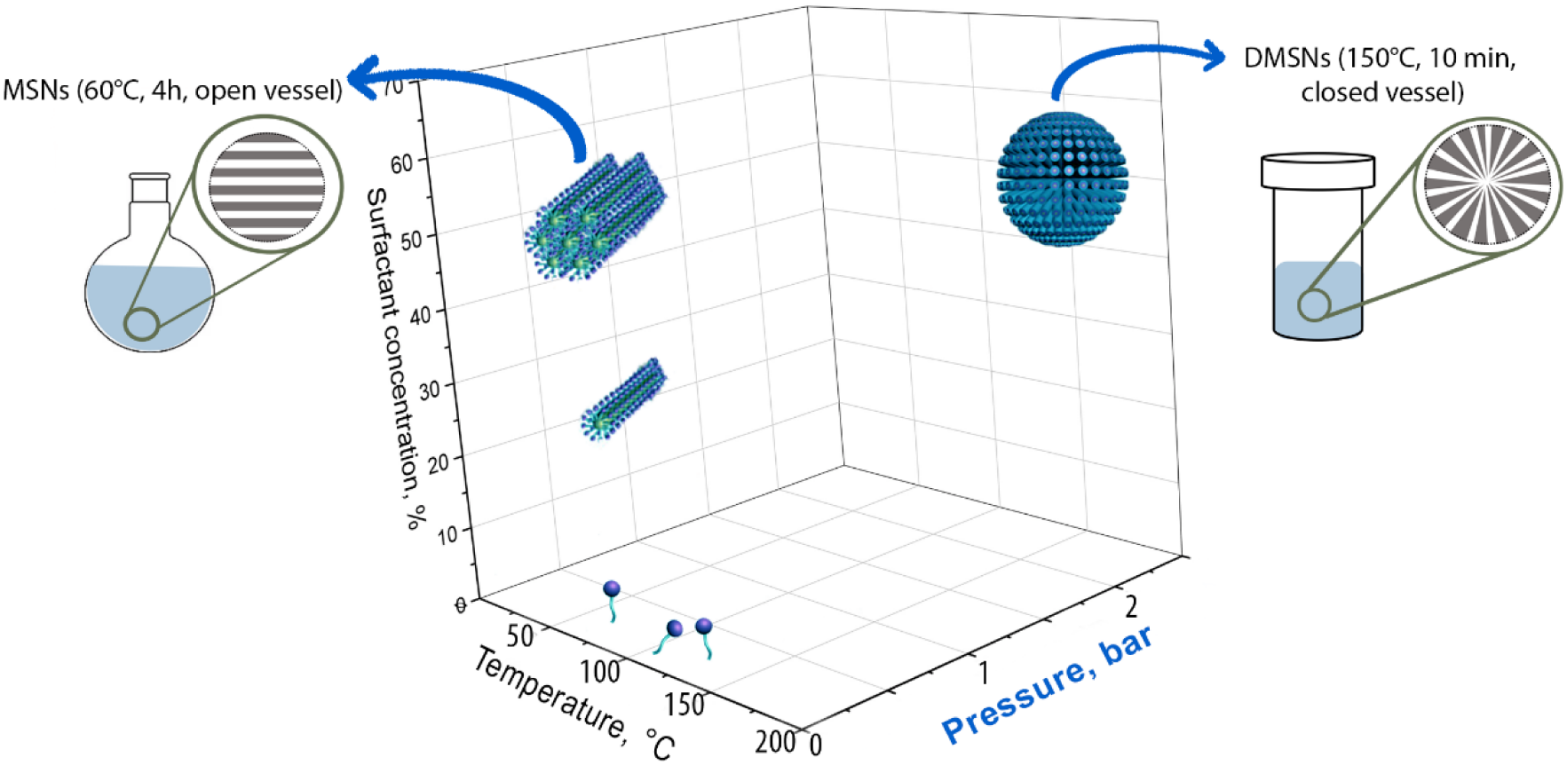
Three-dimensional schematic phase diagram for CTAB in water.

The role of pressure was investigated through a series of experiments conducted at various temperatures and pressures. Further synthesis using a microwave setup were carried out at different pressures, indirectly modulated by varying the total volume in sealed microwave vessels, while keeping the reactant ratios constant. According to the manufacturer’s guidelines, the filling degree was kept within the recommended limit (<60% of the total volume).

Figure SI1, Table SI2, and Figure SI2 report the TEM images, the surface area values, and the size distributions of DMSNs obtained in a total volume of 30 and 50 mL. The internal pressure was estimated based on the combined contributions of the thermal expansion of the trapped air (ideal gas law) and the water vapor pressure at the operating temperature. TEM images show that, in each experimental condition, the nanoparticles obtained exhibit a dendritic channel morphology. Considering the surface area values and size distributions (Table SI2 and Figure SI2), a trend correlated with changes in pressure is evident. Higher filling volumes (50 mL) likely generate increased pressure during microwave heating, promoting faster silica condensation and leading to larger particles with lower BET surface area and pore volume. In contrast, lower filling volumes (30 mL) provide lower-pressure conditions that favor nucleation over particle growth, resulting in smaller nanoparticles with higher surface area and pore volume. Notably, the pore size remains nearly constant (3.2–3.5 nm) across all samples, indicating that pressure mainly influences nanoparticle growth and aggregation.

The critical role of pressure in producing dendritic channel structure was confirmed by performing the first step of the synthesis at 80 °C under atmospheric pressure using a round-bottom flask heated by an external glycerol bath. This step corresponds to the initial phase in which CTAB dissolves in water, self-assembles, and initiates mesopore formation in the presence of the catalyst (NaOH). The synthesis was subsequently completed through the second microwave-assisted step. The resulting materials revealed the absence of a well-defined radial mesoporous texture and the presence of aggregated silica nanodomains. These results suggest that the pressure obtained in the closed tube due to the high temperature is essential for the formation of the characteristic dendritic mesostructure of the DMSNs (Figure SI3b). Moreover, the critical role of high temperatures has been investigated. Indeed, the syntheses performed at lower temperatures (100–110 °C) in either a closed round-bottom flask (Figure SI3c) or a closed microwave vessel (Figure SI3a) do not lead to the formation of the dendritic structure.

This demonstrates that temperatures well above the boiling point of water, along with the relatively high pressure achieved in a microwave setting due to the sealed container, are crucial for obtaining the dendritic structure. It can therefore be concluded that the combination of high temperature and internal pressure directs CTAB micelles to form radial channels during microwave-assisted synthesis in a closed vessel.

### 3.3 Role of Channel Morphology in the Functionalization of Porous Particles

A note on terminology: in the scientific community, the terms “pores” and “channels” are often used interchangeably to refer to cavities within the MSNs. In our work, elongated cavities extending throughout the particle volume are referred to as “channels”, whereas surface openings are designated as “pores”.

The advantages of dendritic morphologies over longitudinal mesoporous structures have long been recognized and demonstrated in applications ranging from catalysis to drug delivery.^24,25^ However, a comprehensive comparison of the two morphologies in terms of functionalization and loading capacity remains poorly explored. In this study, the structural and chemical properties of DMSNs were thoroughly examined through porosity analysis, thermogravimetric measurements, and surface charge determination, revealing a clear enhancement in functionalization capacity relative to conventional MSNs.

The comparison of pore properties was carried out using MSNs and DMSNs synthesized from identical reagents. This approach eliminates potential interferences arising from the use of different chemicals during the purification phase.

TEM imaging, using HAADF-STEM and BF-TEM modes (Figure 3a–d), reveals substantially different channel geometries: an isotropic radial distribution of channels in DMSNs, resembling dandelions, whereas MSNs display hexagonally arranged parallel longitudinal channels. Despite the differences in channel arrangements, both nanoparticle types exhibit a structural periodicity of 3.4 nm, as confirmed by the FFTs in Figure 3a–d and in Figure 3e–f, showing the azimuthal integration of the FFTs obtained from the BF-TEM images. The inner channel diameter, measured from HAADF-STEM images, is approximately 2 nm for both particle types. Due to the radial distribution of channels, the DMSNs expose pores over all their surface, while the MSNs expose pores only at the two opposite sides of the parallel channels.

**Figure 3:**
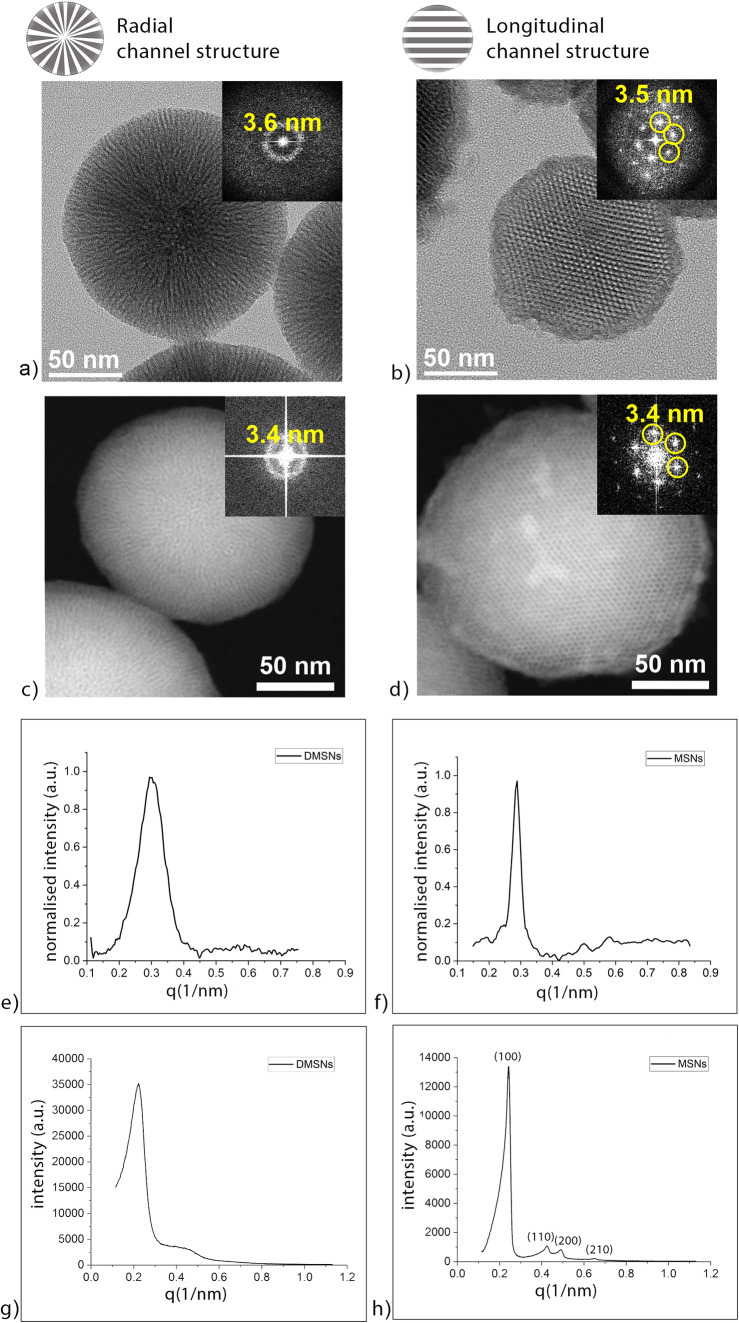
(a) and (b) BF-TEM and (c) and (d) HAADF-STEM images, with corresponding FFTs in the insets, (e) and (f) azimuthal integration of FFTs obtained from Figure 3a–b, and (g) and (h) XRD diffractograms at low angles of DMSNs and MSNs, respectively.

XRD analysis confirms the amorphous nature of silicon oxide in both the DMSNs and MSNs (Figure SI4 in SI). The diffractograms further reveal well-defined reflections characteristic of an ordered two-dimensional hexagonal arrangement of the longitudinal pores, consistent with the p6mm plane group in MSNs, which are absent in the radial pores of DMSNs (Figure 3g and h).

As shown in Table 1, DMSNs demonstrate a markedly higher specific surface area of 1000 m^2^/g compared to 740 m^2^/g for MSNs, along with significantly higher pore volume. According to the BJH analysis, the pore diameters are 3.7 nm for DMSNs versus 3.3 nm for MSNs, and the pore volumes reach 1.1 cm^3^/g and 0.6 cm^3^/g, respectively, highlighting the superior physical properties of DMSNs. The BET values for pore size and volume differ from those obtained by TEM analysis. This can be attributed to the application of the BJH method, which is standardly used for mesoporous materials characterization and does not account for differences in pore morphology. Upon functionalization, the surface areas decrease in both samples, as determined by BET analysis, to 500 m^2^/g for DMSNs and 410 m^2^/g for MSNs. Consequently, the pore size and volume are also reduced after amine functionalization to <2 nm and 0.35 cm^3^/g for DMSN–NH_2_, and <2 nm and 0.33 cm^3^/g for MSN–NH_2_, respectively. As the BJH method is not reliable for pore size determination below 2 nm, to avoid misinterpretation, pore sizes below this threshold are reported as “<2 nm” in Table 1.

**Table 1:** N_2_ Adsorption–Desorption Data of DMSNs and MSNs before and after Functionalisation

	SSA^a^ (m^2^/g)	*V*_t_ ^b^(cm^3^/g)	Pore size (nm)
DMSNs	1000	1.1	3.7
MSNs	740	0.6	3.3
DMSNs–NH_2_	500	0.35	< 2
MSNs–NH_2_	410	0.33	< 2

a Specific surface area.

b Pore volume, and pore size reported as diameter. The BJH model was applied.

This finding is further supported by the thermogravimetric analysis (TGA) and ζ-potential results. In the TGA curve of MSNs (Figure 4b), before the functionalization, a weight loss of approximately 4% around 260 °C is visible. It corresponds to the thermal degradation of residual CTAB^26^ after extensive purification, which is absent from the DMSNs thermogravimetric curve (Figure 4a). This result underscores the challenge of completely removing surfactant from the longitudinal pores of MSNs during the purification phase. In DMSNs, this limitation is not observed, owing to their distinctive pore morphology, which facilitates more efficient surfactant removal. Between 500–550 °C, a weight loss is observed in both DMSNs and MSNs’ TGA curves, corresponding to the decomposition of grafted amino groups. The MSNs show a weight loss of about 9%, while the DMSNs exhibit a higher weight loss of 14%, indicating higher amine content. Another proof of the efficient functionalization of DMSNs was provided by ζ-potential measurements (Figure 4c and d) for both pristine and amine-functionalized DMSNs. They show a more pronounced positive shift for DMSNs-NH_2_, indicating that their surface charge density is consistently higher than that of MSNs.

**Figure 4:**
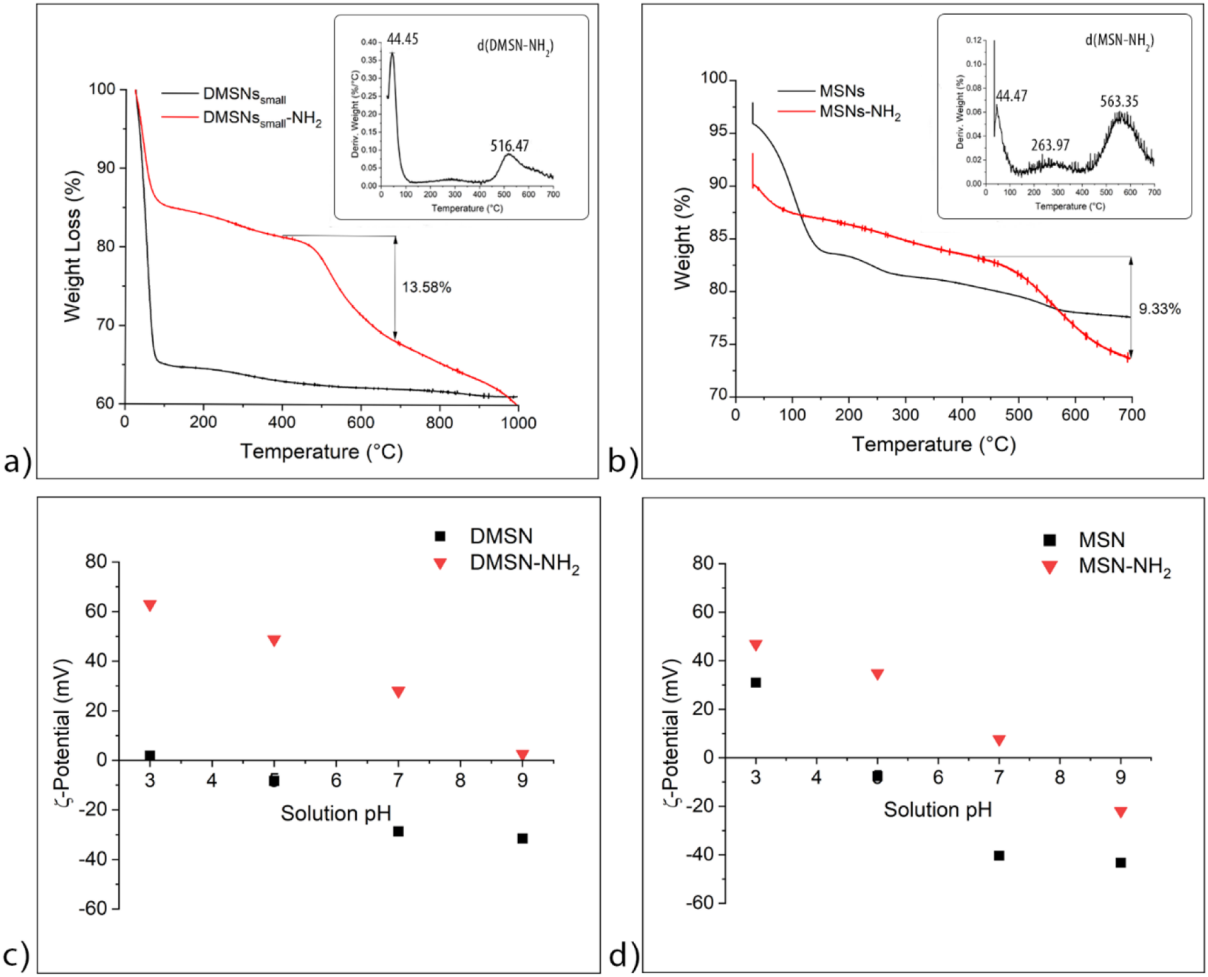
(a) and (b) Thermogravimetric curves and (c) and (d) ζ-potential curves of DMSNs and MSNs, respectively.

To further support this conclusion, the degree of functionalization was quantitatively evaluated from TGA data and normalized to the specific surface area, assuming a propylamine fragment (−(CH_2_)_3_NH_2_, MW = 58 g/mol). The weight losses of 13.58% for DMSN–NH_2_ and 9.33% for MSN–NH_2_ correspond to 2.34 mmol/g and 1.61 mmol/g of amine groups, respectively and when these values are normalized by the BET surface areas after functionalization (500 m^2^/g for DMSN–NH_2_ and 410 m^2^/g for MSN–NH_2_), the resulting amine densities are 0.0047 mmol/m^2^ and 0.0039 mmol/m^2^, respectively.

These multianalytical investigations demonstrate corresponds to an approximately 19% higher surface density of functional groups in DMSNs, demonstrating that the increased functionalization is not solely due to their higher specific surface area but also to a more efficient grafting process.

The radial and open channels of DMSNs facilitate deeper penetration and uniform distribution of functional groups, unlike the linear and parallel channels of MSNs, which are more prone to blockage and, hence, result in lower accessibility.

### 3.4 DMSN–NH_2_ as an Adsorbent for Cobalt Removal from Aqueous Solution

Heavy metal contamination has prompted the development of various remediation technologies, including adsorbents, membrane filtration, and ion-exchange resins, all of which face limitations such as high costs, difficult regeneration, energy demands, hazardous byproducts, and reduced efficiency under variable pH or mixed-metal conditions.^27–28^ Nanomaterial-based adsorbents have emerged as highly promising alternatives.^28–31^ Their large specific surface area and tunable surface chemistry confer enhanced adsorption efficiency, versatility, and more cost-effective regeneration. Moreover, their surfaces can be functionalized with groups such as hydroxyl, thiol, or amino, enabling multiple removal mechanisms, including adsorption, ion exchange, and photochemical separation.

In this study, we evaluated the performance of DMSN–NH_2_ for the removal of Co^2+^ ions from aqueous solutions, demonstrating the potential of DMSN–NH_2_ for water purification applications.^30,32–34^ The removal mechanism is based on the grafting coordination of metal ions to amine functional groups, resulting in stable metal–ligand complexes anchored to the adsorbent surface. In this context, the Co^2+^ removal experiments are intended primarily as a proof-of-concept to highlight potential functional differences associated with pore morphology, rather than to provide a complete performance benchmark.

Both DMSN–NH_2_ and MSN–NH_2_ exhibit rapid initial Co^2+^ adsorption within the first 20 h, followed by a gradual decrease in adsorption rate. As shown in Figure 5a, ICP–OES analysis reveals that DMSN–NH_2_ consistently achieved markedly higher removal efficiency throughout the entire experimental period. Specifically, DMSN–NH_2_ reached approximately 35% of Co^2+^ removal after 20 h. The adsorption capacity (*q*_*t*_)^35^ of DMSN–NH_2_ reached a value of 414 mg/g after 4 h and 694 mg/g at the end of the experiment (144 h). Contrary, MSN–NH_2_ achieved the 15% of Co^2+^ removal, and an absorption capacity of around 16 mg/g after 4 h and 425 mg/g after 144 h. Regarding the notable increase in Co^2+^ uptake by MSN–NH_2_ at longer times, this behavior may be attributed to the gradual intraparticle diffusion of Co ions into the pores. Over time, ions can access previously less accessible −NH_2_ sites located deeper within the mesoporous channels, resulting in a delayed but sustained increase in adsorption. The percentage of cobalt ions remaining in solution during the experiment is shown in Figure SI5.

**Figure 5:**
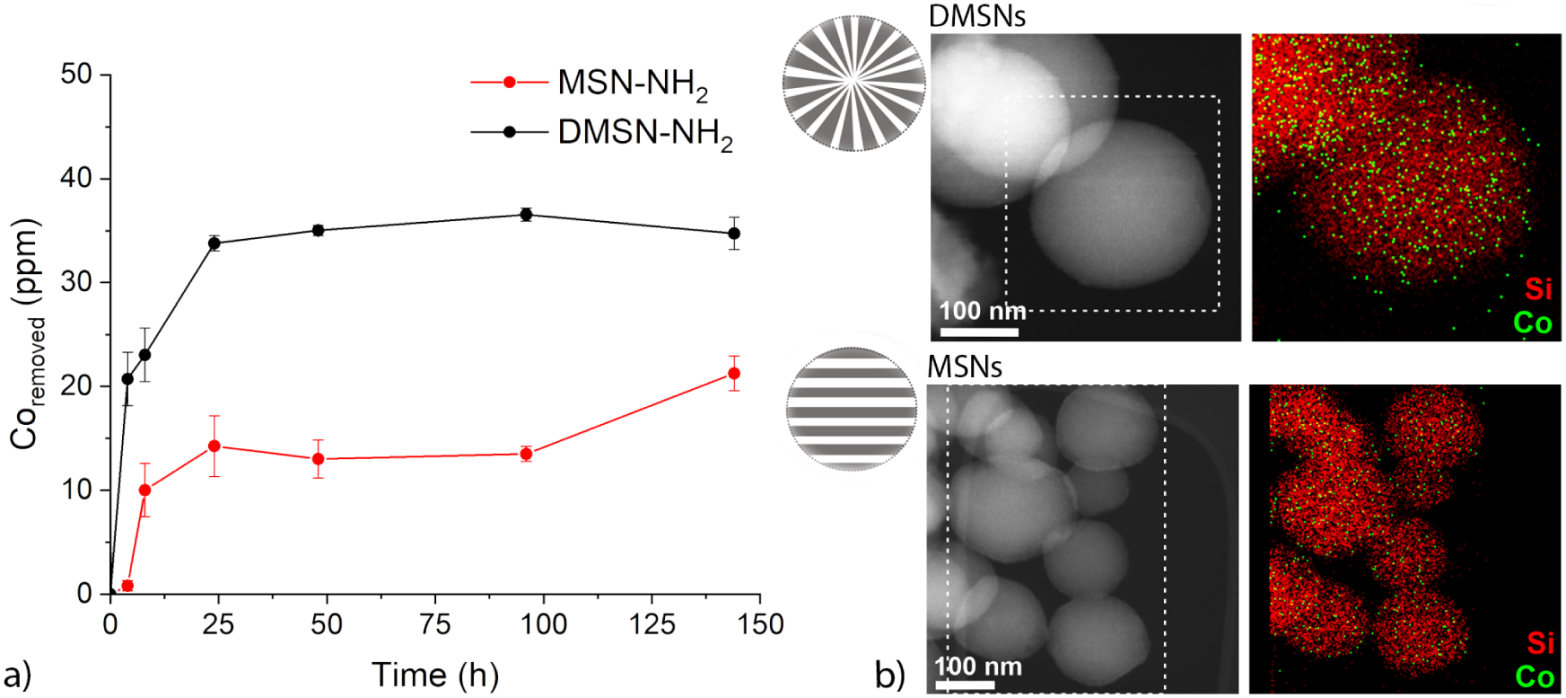
(a) Effect of DMSN–NH_2_ and MSN–NH_2_ on Co^2+^ removal from aqueous solution, and (b) HAADF-STEM and STEM-EDXS maps of Co loading of DMSN–NH_2_ and MSN–NH_2_.

Furthermore, when considering the adsorption performance, the higher uptake of Co^2+^ remains significant even after normalization by the amount of amine groups. These values correspond to 11.78 mmol/g and 7.21 mmol/g of Co^2+^, which, when related to the amine content (2.34 mmol/g for DMSNs and 1.61 mmol/g for MSNs), yield adsorption efficiencies of 5.03 and 4.48 mol Co^2+^ per mol NH_2_, respectively. This indicates an approximately 12% higher efficiency of the active sites in DMSNs. It is worth noting that these values are significantly higher than the theoretical stoichiometric ratio expected for a simple coordination mechanism (2 NH_2_:Co^2+^). This apparent discrepancy highlights that the adsorption process cannot be described solely by direct amine–metal coordination. Instead, the N/Co ratio is influenced by additional contributions such as electrostatic interactions involving protonated amines, surface complexation with silanol groups, and possible ion clustering or precipitation phenomena within the porous network.

Overall, these results indicate that the radial pore architecture enhances not only the accessibility of functional groups but also their effective utilization, leading to improved functionalization and adsorption performance compared to conventional MSNs, beyond what would be expected from stoichiometric considerations alone.

These findings confirm that the dendritic morphology of DMSNs confers superior properties and enhanced functionalization capacity, resulting in significantly improved metal-ion removal performance. In particular, the rapid uptake observed for DMSNs can be attributed to their radial pore architecture. The radial, hierarchically open pore architecture of DMSNs provides shorter diffusion pathways, as pores extend from the core to the external surface, along with higher pore accessibility and reduced tortuosity, thereby facilitating mass transport.^36^ These structural features enable faster and more efficient release kinetics, particularly for larger molecules or in diffusion-limited systems.^37^ Furthermore, the interconnected pore network and wider pore openings in DMSNs may promote more uniform loading and release profiles. In contrast, MSNs with longitudinal channels may exhibit restricted accessibility and predominantly diffusion-controlled release, especially when pore entrances are partially blocked or when strong interactions occur between guest molecules and pore walls.

To further investigate the ion-adsorption mechanism and to demonstrate the role of channel architecture, STEM–EDXS elemental mapping was performed on functionalized nanoparticles after 4 h of exposure to a Co^2+^ solution. As shown in Figure 5b, a uniform cobalt distribution was observed over the nanoparticle surfaces, with DMSN–NH_2_ exhibiting a notably higher cobalt content compared to MSN–NH_2_ (Table SI3 in SI).

While previous studies have reported the potential of DMSNs for heavy metal adsorption,^38^ the majority focus on their integration into hybrid composite systems. Compared with the adsorption capacities reported by Shirazian et al.,^38^ typically ranging from 90 to 1106 mg/g depending on the target ion, functionalization, and experimental conditions, the DMSN–NH_2_ developed in this study demonstrates competitive and, in many cases, superior loading performance.

Importantly, these results highlight that the simplicity of DMSN–NH_2_ enables highly effective Co^2+^ removal without the need for complex, expensive hybrid composites, confirming its potential for water treatment applications. Moreover, the materials can be reused for metal abstraction, as cobalt adsorption is mainly governed by coordination interactions between Co species and surface functional groups. Desorption can be achieved via acid treatment or competitive ligand exchange, which disrupts these coordination bonds and regenerates the active sites.

While the results suggest promising behavior, further studies may be needed to demonstrate a definitive advantage of radial pore architectures for ion adsorption.

## 4 Conclusion

The microwave-assisted, organic-free synthesis described herein demonstrates a highly effective strategy for the rapid, scalable production of DMSNs with a center-symmetric radial channel architecture. This innovative approach addresses and overcomes several critical limitations of conventional synthesis methods by completely eliminating reliance on organic solvents, cosurfactants, and pore-modifying agents, while preserving precise control over particle size, morphology, and porosity. The unique radial channel arrangement of DMSNs not only enhances surface accessibility but also facilitates surfactant removal and improves functionalization efficiency, offering clear advantages over traditional mesoporous silica nanoparticles (MSNs) in terms of performance and versatility.

Beyond these structural and physicochemical benefits, the method’s eco-friendliness and rapidity highlight its sustainability and potential application. The straightforward and reproducible protocol is readily scalable, making it particularly suitable for industrial applications that require large-scale production of well-defined nanomaterials. Collectively, these features position DMSNs as highly promising candidates for a wide range of applications, including heterogeneous catalysis, environmental remediation, drug delivery, and other advanced nanotechnology platforms.

## Supplementary Material



## Notes

Secondary DMSN characterization (DLS, TEM, N_2_ adsorption–desorption, and XRD), as well as figures and tables reporting the complementary experiments we performed to validate the synthesis protocol (PDF)
