# Supplementary material for: Novel, Green, Fast, and Scalable Method for Producing Dendritic Mesoporous Silica Nanoparticles (DMSNs) with High Channel Accessibility
Source: ACS Omega. 2026 Jul 3;11(28):42197–207. doi: 10.1021/acsomega.6c02716 (PMC13393043; doi:10.1021/acsomega.6c02716)
Supplement: Supplementary file 1 [file ao6c02716_si_001.pdf]

Supplementary Information

Novel, green, fast, and scalable method for producing dendritic mesoporous silica nanoparticles (DMSNs) with high channel accessibility

Authors

Roberta Zanini*[a], Sabrina Molinaro[a, b], Luca Leoncino[c], Simone Lauciello[c], Filippo Drago[d], Rosaria Brescia [c], Sergio Marras[e], Elti Cattaruzza[b], Mauro Moglianetti[a], Arianna Traviglia*[a]

Affiliations

[a] Istituto Italiano di Tecnologia-Center for Cultural Heritage Technology, via Adriano Olivetti, 1, 31056 Roncade (TV), Italy

[b] Department of Molecular Sciences and Nanosystems, Ca' Foscari University of Venice, via Torino, 155, 30172, Venice, Italy

[c] Istituto Italiano di Tecnologia, Electron Microscopy Facility, Via Morego 30, 16163, Genoa, Italy

[d] Istituto Italiano di Tecnologia, Chemistry Facility, Via Morego 30, 16163, Genoa, Italy

[e] Istituto Italiano di Tecnologia, Material Characterisation Facility, Via Morego 30, 16163, Genoa, Italy

Table SI1. Overview of synthesis strategies for DMSNs, comparing methodologies[1].

Precursors	Template agents	Catalysts	System	Synthesis methods
TMOS	CTAB	Ammonium hydroxide	*n*-octane-water	Emulsion synthesis method
TEOS	CTAB	Urea	Toluene-*n*-butanol-water	Emulsion synthesis method
TEOS	CPB	Urea	Cyclohexane-pentanol-water	Emulsion synthesis method
TEOS	CTAB	Ammonia	Pentanol-ethanol-water	Emulsion synthesis method
TEOS	CTAB	Urea	Toluene-1-butanol-water	Emulsion synthesis method
TEOS	CTAB	NaOH	Cyclohexane-water	Biphase stratification method
TEOS	CTAB	Ammonia	*n*-hexane-water	Biphase stratification method
TEOS	CTAC	Triethanolamine	Cyclohexane-water	Biphase stratification method
TEOS	CTAC	Triethanolamine	*n*-hexane-water	Biphase stratification method
TEOS	CTAB	Triethanolamine	Cyclohexane-water	Biphase stratification method
TEOS	CTAB+NaSal	Triethanolamine	Water	Homogeneous synthesis method
TEOS	CTAB+SDS	Triethanolamine	Water	Homogeneous synthesis method
TEOS	CTAB	Ammonium hydroxide	Water	Homogeneous synthesis method
TEOS	CTATos	Small organic amines	Water	Homogeneous synthesis method
TEOS	CTAB+FC4	Triethanolamine	Water	Homogeneous synthesis method

[1] S. Zhang, J. Bai, W. Kong, H. Song, Y. Liu, G. Liu, L. Ma, L. Zhou, Y. Jiang, "Dendritic mesoporous silica nanoparticles for enzyme immobilization" *Green Chemical Engineering* **2024**, *5*, 173–186

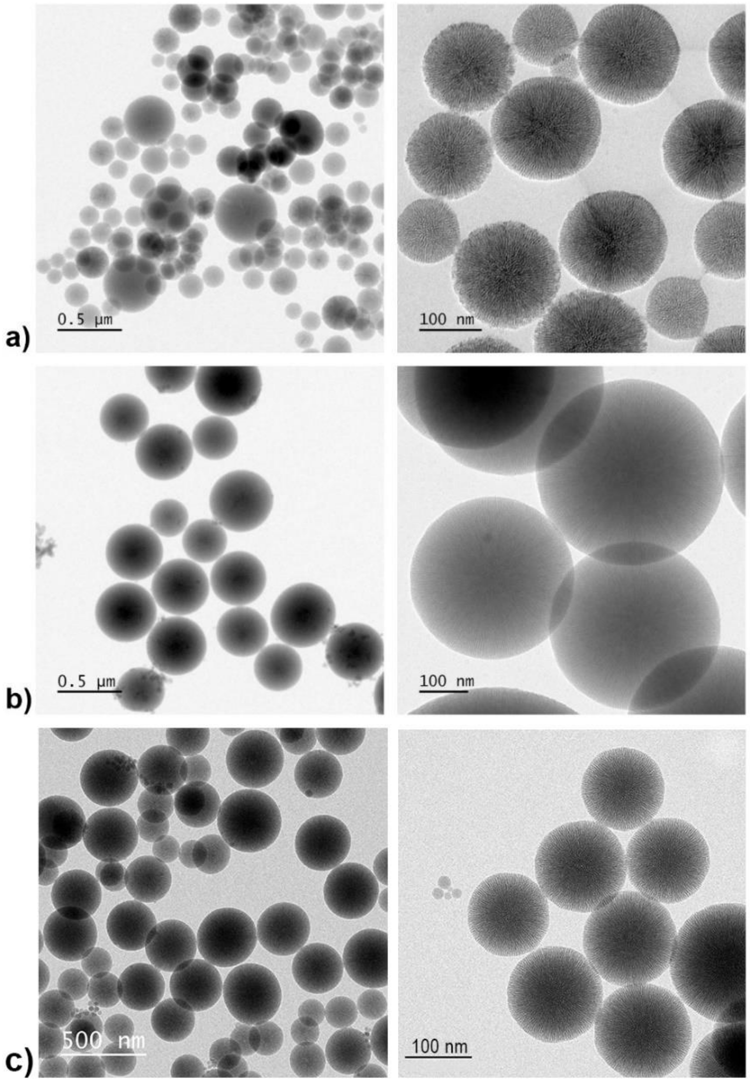

Figure SI1. BF-TEM images of DMSNs synthesis obtained by varying the internal pressure: a)1.66 bar (30 mL of total volume in the microwave tube); b)2.13 bar (50 mL in the microwave tube), and (c) 1.86 bar (as a reference for the 40 mL in the microwave tube).

Table SI2. Overview of the physical properties of the DMSNs obtained by varying the internal pressure.

	Expected pressure at 80°C (bar)	SSA[a] (m^2/g)	V_t^b (cm^3/g)	Pore size (nm)	Average Size (nm)
**DMSNs_V40 mL**	**1,86**	**1000**	**1.1**	**3.7**	**330**
**DMSNs_V30 mL**	1,66	1130	1.3	3.5	270
**DMSNs_V50 mL**	2,13	811	0.8	3.1	690

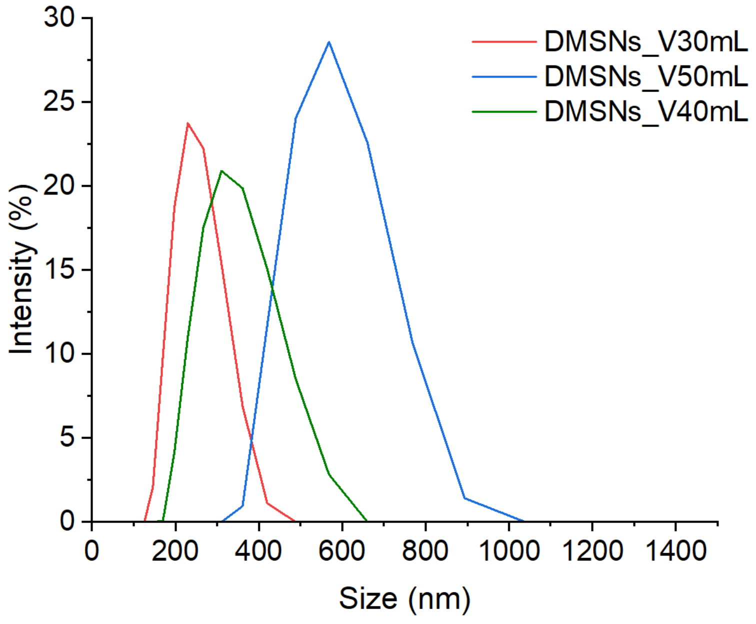

Figure SI2. Size distribution of DMSNs synthesis obtained by varying the internal pressure.

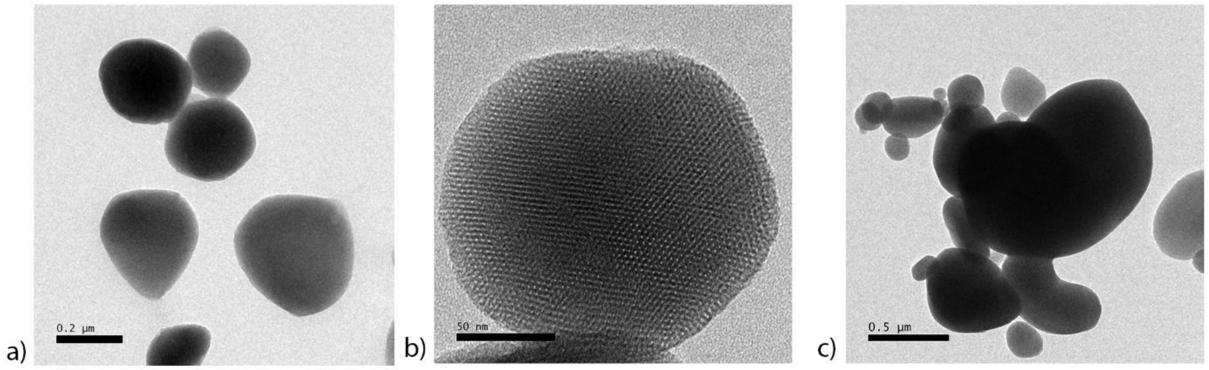

Figure SI3. BF-TEM images of DMSNs synthesis performed at (a) 100°C in microwave tubes, (b) at 80°C under atmospheric pressure for the dissolution and self-assembly of CTAB in water, followed by the subsequent microwave-assisted step as described in the protocol, and (c) at 110°C in a closed round-bottom flask.

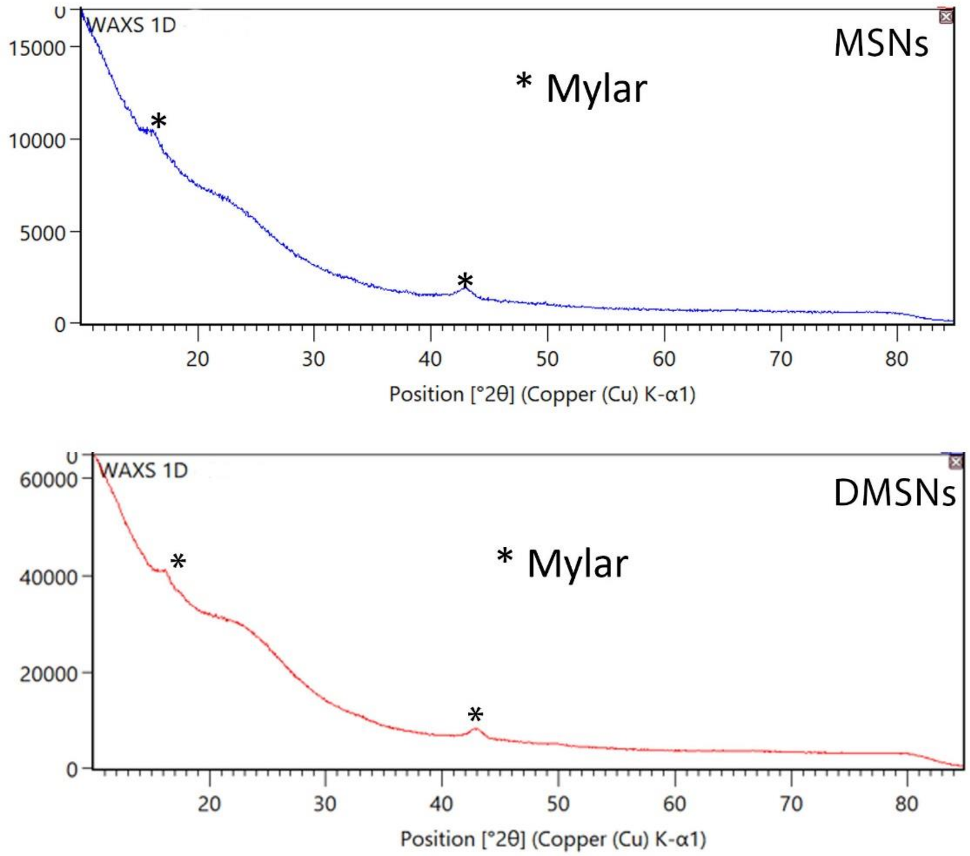

Figure SI4. XRD diffractograms of MSNs and DMSNs at high angles.

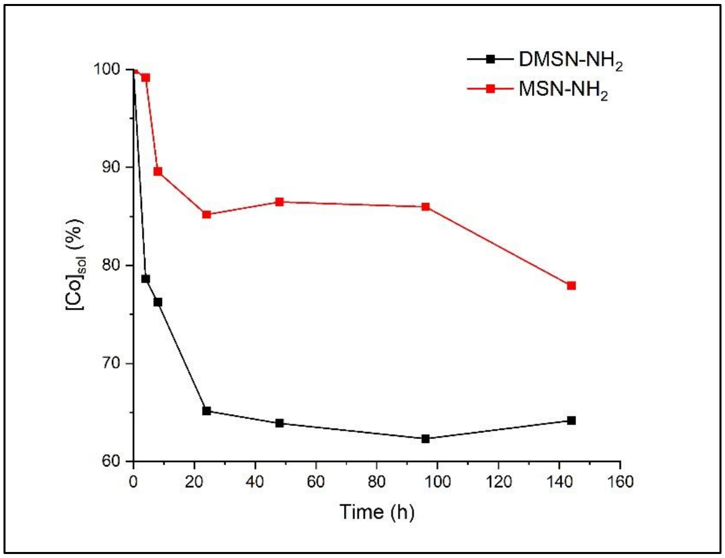

Figure SI5. Percentage of Co ions remains in solution during the ion removal experiment.

Table SI3. Quantitative data obtained from STEM-EDS analysis and ICP-OES.

**Samples**	**Co/Si molar ratio (STEM-EDS)**	**Co/Si molar ratio (ICP-OES)**
**MSNs**	0.014	0.017
**DMSNs**	0.026	0.058
